# Assessment of client satisfaction on emergency department services in Hawassa University Referral Hospital, Hawassa, Southern Ethiopia

**DOI:** 10.1186/s12873-017-0132-7

**Published:** 2017-06-27

**Authors:** Mesfin Worku, Eskindir Loha

**Affiliations:** Department of Medical Laboratory Sciences, Hawassa University, College of Medicine and Health Sciences, Hawasa, Ethiopia

**Keywords:** Patient satisfaction, Ethiopia, Emergency department

## Abstract

**Background:**

Satisfaction refers to a state of pleasure or contentment with an action, event or service, especially one that was previously desired. Regarding to client, satisfaction is the level of happiness that clients experience having used a service. It therefore reflects the gap between the expected service and the experience of the service**,** from the client’s point of view. Information was unavailable regarding the level of satisfaction of patients towards emergency health care servicesat Hawassa University Referral Hospital thatserve a huge catchment area; and this study addressed this gap.

**Method:**

Cross-sectional study was conducted from March 13 to May 15/2014. Systematic sampling method was used to enroll study participants. The data was collected by trained data collectors using pre-structured questionnaire.

**Results:**

A total 407 clients were enrolled under this study with respondent rate of 96.9%. Nearly two third of study participants were male, 270 (66.3%). 86.7% of study participants were satisfied by services provided in Emergency Out Patient Department (OPD). The percentage of study satisfaction with physical examination by Doctor, nursing, laboratory and pharmacy services were 95.6%, 89.9%, 84.7% and 67.6%, respectively. Only 31.9% were satisfied with availability of drugs in the pharmacy. Regarding to staff courtesy, 91.7% of study participants were satisfied by the manner shown by the staffs working in Emergency OPD. The vast majority of the participants (97%) were satisfied with the courtesy of Medical laboratory personnel and the least satisfaction (79.4%) was recorded for security guards.

**Conclusion:**

This study showed low level of patient satisfaction in pharmacy services specifically due to unavailability of drugs due to lack of sustained supply of drugs.

**Electronic supplementary material:**

The online version of this article (doi:10.1186/s12873-017-0132-7) contains supplementary material, which is available to authorized users.

## Background

Satisfaction refers to a state of pleasure or contentment with an action, event or service, especially one that was previously desired [1]. Regarding to client, satisfaction is the level of happiness that clients experience having used a service. It therefore reflects the gap between the expected service and the experience of the service**,** from the client’s point of view [2]. Furthermore, patient satisfaction is the patient’s perception of care received compared with the care expected [3].

One of the WHO’s six building blocks of health systems is the delivery of health services that are effective, safe and of good quality for those who need them [4]. At a hospital level, providing a quality service is usually challenged by burdensome patients’ flow and the urgent nature of care in the emergency department(ED)further suppresses the effort. And hence, assessing the patients’ satisfaction as a quality of care indicator is required to monitor the non-technical aspects of quality of care in such settings [5].

Measuring client or patient satisfaction has become an integral part of hospital/clinic management strategies across the globe [6]. Moreover, the quality assurance and accreditation process in most countries requires measuring the satisfaction of clients on a regular basis [7]. Moreover, patient satisfaction had been an important issue for health care managers and health care providers [8]. Among factors influencing patient satisfaction, the relationship between health care providers and patients was reported to be the most influential [9]. Meanwhile, expectation about the services, perceived adequacy of consultation duration, welcoming approach and perceived body signaling are considered as determinants of satisfaction [10].

As patient satisfaction is considered to be a health care outcome and predictor of treatment utilization and adherence to the care and support, assessment of the level of patient satisfaction is very vital. In addition, knowing the needs of patient is of paramount essential for the achievement of sustainable development goal on health service delivery [11]. Since there is no study on patient satisfaction related to emergency department health care service at Hawassa University Referral Hospital, the current study aimed to assess the level of patients’ satisfaction on emergency department health care services.

Ethiopia has a three-tier health system: primary, secondary and tertiary level health care. The primary level health care is composed of health post, health center and primary hospital. A general hospital constitutes the secondary level health care. Meanwhile, Specialized Hospital makes the tertiary level and expected to serve up to five million people. And this study was conducted at a facility grouped under tertiary level care [12].

## Methods

### Study setting

Hawassa is the capital city of southern Ethiopia and it is located 275 km to the south of the capital city of Ethiopia, Addis Ababa. Hawassa University Referral Hospital (HURH) is a tertiary level hospital serving around 12 million people in the Southern Nations, Nationalities and People Region (SNNPR) and the neighboring Oromia region.

### Study design and study period

Across-sectional study was conducted from March 13 to May 15/2014.

### Sample size determination and sampling method

Sample size was determined using a single population proportion formula. A 95% confidence level, 5%margin of error and 54.1% anticipated satisfaction level of the patients were considered as inputs [5]. Systematic sampling method was employed. Busy work hours, shifts, personnel, different providers, day of the week and type of client complaint were considered to have had an effect on satisfaction level. The total sample size was distributed to different shift proportionately. In order to select participants in each shift, random numbers were used.

### Measurement and data collection

Pre-structured questionnaire were developed by authors for current research in English and then translated to working language of country (Amharic). Data was collected via face-to-face interviews by trained data collectors rather than investigator to avoid researcher biases. The questionnaire contained satisfaction indicators socio-demographic characteristics of the emergency clients and different dimensions of emergency services such as consultation time with physician, courtesy of staff, health care service, and waiting time (Additional file 1).

Due to the fact that emergency service is given to 24 hours only, the clients were interviewed immediately after getting emergency service within this time frame. i.e. at the time of admission to inpatient ward from emergency department or before the clients go to their home after getting emergency service.

### Quality control

To maintain the quality of the data, data collectors were trained and the questionnaire was pretested. The interviewers did not wear uniforms or badges. The interviewers were oriented about unifying their communication and the process of interviewing the clients.

### Data analysis

Data was cleared, edited, coded after it was entered into Epi Info version 3.5.1 and was exported to SPSS version 20. Descriptive statistics was used to determine satisfaction indices. Factor analysis was done to identify factors that explained most of the variance observed in the population with regard to each scale.

### Operational definition

Clients: are individuals who are patients, relatives and friends get services directly or indirectly from emergency department.

Relatives or friends: individuals who brought the patient and responds about service while they accompany the patients.

Urban: refers to Hawassa town where clients is living.

Rural: refers to out of Hawassa town where clients.

## Results

A total 407 clients were enrolled under this study *with respondent rate of 96.9%*. Nearly two third of study participants were male 270 (66.3%). The age of the study participants ranged betweenwas18 and 67 years. The majority of study subjects were in age group 28–37 years. Regarding the level of education, high school students (31.7%) outnumbered the rest. The study participants were getting services in three shifts: morning, evening and night. The busiest time of visit was morning (47.9%), which was followed by evening (40.0%) and night (12.0%). Nearly half, 47.2% of the respondents visited the emergency department for the first time. Concerning to the address of study participant, slightly more than half of the participants were from urban areas, 242 (59.5%). The vast majority of the interviewees (91.4%) were relatives/friends (Table 1).

**Table 1:** The sociodemographic characteristics of study participants of emergency outpatient department, March 13 to May 15/2014, Hawassa University Referral Hospital, Hawassa, southern Ethiopia

Demographic characteristics	Frequency	Percent
Gender		
Male	270	66.3
Female	137	33.7
Total	407	100.0
Age		
18-27	98	24.1
28-37	140	34.4
38-47	116	28.5
48-57	49	12.0
58-67	4	1.0
Total	407	100.0
Level of education		
Illiterate	27	6.6
Read and write	55	13.5
Elementary	60	14.7
High school	129	31.7
Diploma	83	20.4
Degree and above	53	13.0
Total	407	100.0
Timeofvisit		
Morning	195	47.9
Evening	163	40.0
Night	49	12.0
Total	407	100.0
Frequency of visit		
New	192	47.2
Repeat	215	52.8
Total	407	100.0
Client		
Patient	35	8.6
Relative/friend	370	91.4
Total	407	100.0
Address of client		
Urban	242	59.5
Rural	165	40.5
Total	407	100.0

The majority, 86.7% of the study participants were satisfied by services rendered in emergency department. The satisfaction rate in in the professional care rendered by doctors, nurses, laboratory and pharmacy personnel were 95.6, 89.9, 84.7 and 67.6, respectively. The least satisfaction rate, 31.9% was specifically reported about the availability of drugs in the pharmacy. Regarding to staff courtesy, 91.7% of study participants were satisfied by the manner shown by staffs and among these 97% were satisfied by laboratory personnel, and the lowest satisfaction rate (79.4%) was recorded for courtesy of the hospital guards (Table 2).

**Table 2:** The overall satisfaction rate of study participant regarding services and courtesy of staff at emergency outpatient department, from March 13 to May 15/2014, Hawassa University Referral Hospital, Hawassa, southern Ethiopia

Questions	Dissatisfied (%)	Satisfied (%)
Consultation time with physician	19(4.7)	383(95.3)
Courtesy of staff		
Doctor	19(4.7)	387(95.3)
Nurse	28(6.9)	377(96.1)
Medical Laboratory professionals	12(3.0)	376(97.0)
Pharmacy professionals	35(8.7)	366(91.3)
Registration staff	25(6.2)	380(93.8)
Securityguard	84(20.6)	323(79.4)
Porter	29(7.4)	364(92.6)
Casher	34(8.6)	363(93.4)
The overall satisfaction of courtesy	32(8.3)	367(91.7)
Health care service		
Physical examination by Doctor	18(4.4)	388(95.6)
Nursing services	41(10.1)	363(89.9)
Medical Laboratory services		
Availability of the test	94(24.3)	293(76.6)
Waiting time to get the lab professionals	21(5.5)	364(94.5)
Waiting time to get lab result	32(8.3)	353(91.7)
Payment for lab tests.	65(17.0)	318(83.0)
Cleanliness of lab	76(19.8)	308(81.2)
Cleanness of Waiting area	99(25.7)	286(74.3)
Completeness of information	25(6.5)	360(93.5)
The overall satisfaction of Lab services	59 (15.3)	326(84.7)
Pharmacyservice		
Availability of drug	270(68.1)	127(31.9)
Payment for drug	79(20.2)	313(79.8)
Completeness of information on how and when to take the prescribed drugs	34(8.8)	361(91.2)
The overall satisfaction of pharmacy services	128(32.4)	267(67.6)
The overall satisfaction of ED services	53 (13.4)	345(86.6)

*ED* Emergency Department

## Discussion

In Ethiopia, the health care insurance scheme is on its early stage and the implementation is yet to be initiated. And none of the clients who visited this hospital had any kind (private/government) of health insurance and all were paying out of their pocket for the service. Clients visiting the emergency department have the same socio-economic profile with that of the general population of the country since this hospital is the first of its kind in the region in terms of providing tertiary/specialized level care. Meanwhile, quality service provision is expected, service rendered in emergency department needs greater emphasis.. Therefore, factors affecting this service should be identified and necessary measures should be taken to improve the quality of the service [13].

Our study showed that the overall patient satisfaction level of service given in Emergency OPD was 86.7%. This is higher than study conducted in Eastern part of Ethiopia (54.1%), South Western part of Ethiopia (77.0%) and Tertiary Hospital in south Nigeria (66.8%) [2, 5, 8]. This variation may be attributed to the fact that this study was conducted in a high-level facility with relatively sufficient number of health professional. In addition, differences in the time of the study and the type the study participants may be another reason for the variation.

The current study indicated that 95.3% of study participants were satisfied with physical examination by the physicians. This finding is slightly greater than the study conducted at Jimma University specialized hospital and Tertiary Hospital in south Nigeria [5, 8]. However, a recent study from Jimma specialized hospital indicated that the satisfaction level was much lower (60.3%) than this study [14].

Regarding the service provided by the nursing staff, 89.9% of the participants were satisfied with the care. This study finding is comparable with study conducted in Black Lion Hospital (90.1%), Addis Ababa, Ethiopia [15]. Similar study from Iran [13] reported that 82.8% of the study participants were satisfied and, which is a little bit lower than this figure in the current study. On the other hand, the report (67.1%) from Gondar (Ethiopia) [16] was much lower. The probable reason may be the nature of the clients (the level of satisfaction by the patients themselves and their relatives could be vary), differences in the level of facilities and also time.

Concerning pharmacy services, 68.1% of study participants were dissatisfied. This was mainly because of the unavailability of drugs partially or totally in the unit. The stock-out might be related to the government drug supply system that was lengthy for the sake of averting corrupted practices. The complaints of the clients we observed were due to the unavailability of the item while they were prepared to purchase out of pocket.As a result the clients were forced to buy prescribed drugs with exaggerated price from private pharmacies. Because of this fact, more than half of the study participants were dissatisfied by pharmacy services. This finding is comparable with a similar study conducted in Jimma specialized hospital that reported 70% satisfaction level [2, 7].

One of the domains of emergency outpatient department is laboratory service. Based on the findings of the current study, the overall satisfaction rate of laboratory service was84.7%. This result is comparable with the report (85.5%) of similar study conducted in Addis Ababa, Ethiopia [17]. However this finding is higher than the report (60.4%) of other Ethiopian study from Nekemte Referral Hospital, [18].

Based on current study, 91.7% of study participants were satisfied by the courtesy of the staffs working in Hawassa University Referral Hospital Emergency OPD. However the satisfaction rate of study participants about their encounter with security guards was relatively low compared to the study conducted in Iran [13]. The differences might be due to misunderstanding of manners, duties and responsibilities of clients’ handling.

### Limitations

Evidence based intervention related to client satisfaction can be implemented based on such assessment data. However we believe that there may bepotential confounding factors that we did not take into account neither in data collectiontools preparation nor in the analysis. We didn’t measure waiting time from patient arrival until leaving the emergency outpatient department. Patients with different clinical presentations might have different satisfaction rates, and also the severity of cases may influence satisfaction rates. On the other hand using a relative or friend accompanying the patient to assess satisfaction of the service (the majority in our case) might have its own limitations since their view could be different from the patient him/herself. In addition, the absence of other alternative tertiary level care facility may suppress disclosing dissatisfactions and also compel the respondents to submit to the standards of care without questioning. In addition, there are no standard procedures or tools to assess patient satisfaction in Ethiopian hospitals as in the case in developed nations. In addition, the hospital does not have its own mechanism of assessment in a structured way but there is an office to hear complaints (if any) and try to solve on the spot. We did this assessment taking into account the experience of other researchers.

## Conclusions

The lowest level of patient satisfaction (31.9%) was recorded in pharmacy services specifically in relation to unavailability of drugs. Similarly, rate of satisfaction with the courtesy of the hospital guard, availability of the tests and cleanness of the waiting area was 79%, 76.6% and 74.3%, respectively. Therefore, the authors recommend that the hospital administration may need to take the necessary action on the gaps identified.

## Additional file

Additional file 1: Questionnaire. (DOCX 16 kb)

## Abbreviations

ED: Emergency department
EL: Eskindir Loha
HURH: Hawassa University Referral Hospital
IRB: Institutional Review Board
MW: Mesfin Worku
OPD: Outpatient department
SNNPR: South Nation Nationalities People Region
WHO: World Health Organization
