# Supplementary material for: Assessment of client satisfaction on emergency department services in Hawassa University Referral Hospital, Hawassa, Southern Ethiopia
Source: BMC Emerg Med. 2017 Jun 27;17:21. doi: 10.1186/s12873-017-0132-7 (PMC5488300; doi:10.1186/s12873-017-0132-7)
Supplement: Additional file 1: — Questionnaire. (DOCX 16 kb) [file 12873_2017_132_MOESM1_ESM.docx]

**Annex-1**

**Hawassa University**

**College of Medicine and Health Science**

**Department of Medical Laboratory Science**

**Questionnaire on assessment of patient satisfaction in emergency department of Hawassa University referral Hospital.**

1. **Demographic characteristics**
2. What is your gender?

Female

Male

1. Age

18-27

28-37

38-47

48-57

58-67

1. What is your Level of education?

Illiterate

Read and write

Elementary

High School

Diploma

Degree and above

1. Please indicate your time of visit?

Morning

Evening

Night

1. Is it the first visit to this Hospital?

Yes

No

1. Who has completed the questionnaire

Patient

Another one

1. Where are you come from?

Urban

Rural

1. Question on waiting time before getting service

< 30 minutes > 30 minutes

For physician

For Nurses

For pharmacist/druggist

For laboratory professionals

For registration

For casher

1. **Satisfaction level of clients**

| **No** | **Questions** | **Very satisfactory** | **Satisfactory** | **Dissatisfactory** | **Very dissatisfactory** |
| --- | --- | --- | --- | --- | --- |
| 1 | Consultation time with physician |  |  |  |  |
| 2 | Courtesy of staff |  |  |  |  |
|  | - Doctor |  |  |  |  |
|  | - Nurse |  |  |  |  |
|  | - Laboratory professional |  |  |  |  |
|  | - Pharmacy professional |  |  |  |  |
|  | - Registration staff |  |  |  |  |
|  | - Guards |  |  |  |  |
|  | - Porter |  |  |  |  |
|  | - Casher |  |  |  |  |
| 3 | Health care service |  |  |  |  |
|  | - Physical examination by Doctor |  |  |  |  |
|  | - Nursing services |  |  |  |  |
|  | - Laboratory services |  |  |  |  |
|  | - - Availability of the test |  |  |  |  |
|  | - - Waiting time to get the lab professionals |  |  |  |  |
|  | - - Waiting time to get lab result |  |  |  |  |
|  | - - Payment for lab tests. |  |  |  |  |
|  | - - Cleanliness of lab |  |  |  |  |
|  | - - Waiting area |  |  |  |  |
|  | - - Completeness of information on how and when to receive lab result |  |  |  |  |
|  | - Pharmacy service   - Availability of drug |  |  |  |  |
|  | - - Payment for drug   - Completeness of information   on how and when to take the prescribed drugs |  |  |  |  |
